# Cancer-related health behaviours of young people not in education, employment or training (‘NEET’): a cross-sectional study

**DOI:** 10.1186/s12885-017-3157-0

**Published:** 2017-03-02

**Authors:** Catherine H. Stewart, Philip Berry, Dunja Przulj, Charlene Treanor

**Affiliations:** 1 MRC/CSO Social & Public Health Sciences Unit, University of Glasgow, Top Floor, 200 Renfield Street, Glasgow, G2 3QB UK; 2 Northern Institute for Cancer Research, Newcastle University, Framlington Place, Newcastle, NE2 4HH UK; 3 Health & Lifestyles Research Unit, Wolfson Institute of Preventive Medicine, Queen Mary University of London, 2 Stayner’s Road, London, E1 4AH UK; 4 Centre for Public Health, School of Medicine, Dentistry and Biomedical Sciences, Queen’s University Belfast, Institute of Clinical Sciences B, Royal Victoria Hospital Site, Grosvenor Road, Belfast, BT12 6BJ UK

**Keywords:** NEET, Cancer, Health behaviours, Young adults, Unemployed, Smoking, Alcohol, Exercise, BMI, Lifestyle

## Abstract

**Background:**

Links between participating in unhealthy behaviours, e.g. smoking, and an increased risk of developing some cancers are well established. Unemployed adults are more likely to participate in cancer-related health behaviours than their employed counterparts. However, evidence of whether this is true in young adults not in education, employment or training (NEET) compared to their ‘non-NEET’ peers is either limited or inconclusive. Using cross-sectional health data from across the UK, this study aims to investigate whether participation in cancer-related health behaviours varies by NEET status.

**Methods:**

Data for 16–24 year olds were extracted from the 2010–12 Health Surveys for England (HSE) and Scottish Health Surveys (SHeS). Information on economic activity in the last week was used to determine NEET status. Data on whether respondents had been seeking employment within the last four weeks and availability to start within the next two weeks allowed NEETs to be further identified as unemployed (UE) or economically inactive (EI). Logistic regression modelled the effect of being NEET on odds of being a current smoker; heavy drinker; not participating in sport; having eaten less than five portions of fruit or vegetables the day before survey interview and having an unhealthy body mass index (BMI). Analyses were performed before and after exclusion of EI NEETs.

**Results:**

Data were extracted for 4272 individuals, of which 715 (17%) were defined as NEET with 371 (52%) and 342 (48%) further classified as UE and EI respectively. Two NEETs could not be further defined as UE or EI due to missing information. Relative to non-NEETs, NEETs were significantly more likely to be current smokers, not participate in sport and have an ‘unhealthy’ BMI. These results held after adjustment for socio-demographic characteristics both before and after exclusion of EI NEETs. Before exclusion of EI NEETs, NEETs were significantly less likely to be heavy drinkers than non-NEETs. There was no significant difference in likelihood of heavy drinking between NEETs and non-NEETs when excluding EI NEETs.

**Conclusions:**

NEETs were generally at an increased risk of participating in cancer-related health behaviours than non-NEETs. As the likelihood of becoming NEET is greater in socioeconomically-disadvantaged groups, interventions to discourage unhealthy behaviours in NEETs may contribute to a reduction in health inequalities.

## Background

Young people are defined as ‘NEET’ if they are aged 16–24 years old and Not In Education, Employment or Training (NEET) [1]. In the second quarter of 2016 there were an estimated 865,000 ‘NEETs’ in the United Kingdom (UK) [2]. Research has demonstrated both medium and long-term economic effects of becoming NEET at time of school-leaving with such individuals being more likely to still be unemployed up to five years later as well as being at an increased risk of being unemployed or in a low-paid job up to ten years later [3]. NEETs who do eventually find employment are more likely to face a lifetime of poorer income [4], lower social class [5] and lower levels of job satisfaction [6].

However, consequences of being NEET are not restricted to poorer economic outcomes. Unemployment at younger ages has been demonstrated to have immediate adverse effects on health including increased rates of poorer mental wellbeing [7], depression [8] and suicidal behaviours [9] amongst those who are NEET. Moreover, limited research has also shown some negative effects of unemployment at younger ages on long-term health. Functional somatic symptoms [10], chronic limiting illness [5] and psychological symptoms [11] in adulthood are all reported to be consequences of youth unemployment.

The association between unemployment and poor health can be explained somewhat by increased participation in unhealthy behaviours, such as smoking and drinking alcohol amongst unemployed individuals [12, 13]. However, the evidence on whether participation in unhealthy behaviours among NEETs is greater than among their ‘non-NEET’ peers is either limited or inconclusive [14]. Whilst some studies have reported significant associations between NEET status and smoking [7], Baggio et al. [15] found that although smoking was likely to increase the risk of becoming NEET, the pathway from NEET status to tobacco use was not significant. Similarly, significant associations between being NEET and increased drinking or alcohol abuse/dependence have been found in some studies [7, 9], but not in others [14]. Additionally, the correlation between unemployment and increased alcohol consumption found by Janlert and Hammarström [16] only applied to longer periods of unemployment. There have also been reports of lower levels of involvement in sport or exercise amongst NEETs [17].

Participation in unhealthy behaviours has been linked to an increased risk of developing a range of cancers [18, 19]. In 2012, the most common cancers in Europe, representing half of the overall burden of cancer, were breast, colorectal, prostate and lung cancer [20]. Previous research has attributed some of the risk of developing each of these four cancers to participation in unhealthy behaviours including smoking (lung) [21], alcohol consumption (breast) [22], low fruit and vegetable intake (lung, colorectal) [23], physical inactivity (breast, colorectal, prostate) [24] and excess body weight (colorectal) [25]. Given the association between unemployment and increased participation in unhealthy behaviours and well-established links between participating in such behaviours and cancer, NEETs may be at an increased risk of cancer. Although links between unemployment and cancer have been shown to exist [26, 27], studies have either tended to focus on unemployment in middle age or use cohorts spanning a wide range of ages. Studies focusing primarily on unemployment in early adulthood as a risk factor for cancer and which also cover the other dimensions included in the NEET definition, i.e. not in education or training, are lacking. This study aims to develop such an evidence base by investigating whether NEETs have higher rates of participation in cancer-related health behaviours compared to non-NEETs.

## Methods

### Aims of the study

Using cross-sectional health survey data for samples of 16–24 year olds, the aims of this study were: (i) to compare socio-demographic and mental and physical health-related characteristics of NEETs and non-NEETs; (ii) to investigate whether participation in cancer-related health behaviours were greater amongst NEETs and; (iii) whether any association between NEET status and such health behaviours persisted even after adjustment for socio-demographic and mental and physical health-related factors.

### Design & setting of the study

Data for all 16–24 year olds who participated in the Scottish Health Survey (SHeS) and Health Survey for England (HSE) over the years 2010–2012 were downloaded from the UK Data Service [28–33]. The SHeS and HSE were designed to provide nationally-representative samples of adults (aged 16 years and over) and children (aged 0–15 years) in the general population living in private households in Scotland and England. Both were based on a two-stage stratified random sample design. Postcode sectors in each constituent country were ordered by region (Health Board in Scotland and Local Authority in England) and deprivation. The first stage of the design involved creating a sample of randomly-selected postcode sectors. At the second stage, a sample of addresses was randomly drawn from each selected postcode sector based on the Postcode Address File (PAF). All adults and up to two children at each address were eligible for inclusion in the survey. If there were more than two children within a household, then two were randomly selected for inclusion [34–37]. The health surveys were chosen as they contained data on a wide range of socio-demographic variables, including economic destination of respondents, as well as information on cancer-related health behaviours. Using data from Scotland and England provided a more representative view of NEETs across the UK and allowed for testing of independent effects of each constituent country on health outcomes.

### Health behaviour outcomes

Binary indicator variables (yes/no) were created to reflect the following cancer-related health behaviours: current smoker; heavy drinker (defined as >14 units of alcohol per week for females and >21 units for males); participation in sport; <5 portions of fruit/vegetables the day before survey interview and unhealthy BMI. Unhealthy BMI referred to ‘underweight’ (BMI <18.5 kg/m^2^), overweight (BMI 25–29.99 kg/m^2^) or obese (BMI > =30 kg/m^2^).

### NEET status

Survey data on economic activity in the last week were used to create a NEET indicator variable. Respondents were defined as ‘NEET’ if activity included the following: unpaid work for their own or a relative’s business; waiting to take up paid work; looking for paid work or a government training scheme; intending to seek work, but temporarily sick or injured; permanently unable to work or looking after home or family. The UK Government further classifies NEETs into unemployed or economically inactive [1]. NEETs are defined as unemployed (UE) if they have been actively seeking work within the last 4 weeks and would be available to start work within the next two weeks [1]. Otherwise, NEETs are defined as economically inactive (EI) if they have not been seeking work within the last 4 weeks and/or would not be able to start work within the next two weeks [1]. The EI definition captures long-term sick/disabled individuals or individuals looking after family/children. Survey data contained information on whether respondents had been seeking employment within the last 4 weeks and whether they would be available to start within the next 2 weeks, thus allowing a variable to be created to further identify NEETs as UE or EI.

### Socio-demographic & health-related characteristics

Information obtained from survey data included sex, age, ethnicity, marital status, car/van access, top academic qualification, housing tenure, receipt of means-tested benefits, total annual household income and a measure of socio-economic position using the National Statistics Socio-economic classification (NS-SEC) [38]. Measures of physical and mental health status were also available including limiting long-term illness, self-assessed general health and non-psychiatric morbidity assessed using the 12-item General Health Questionnaire (GHQ-12) [39]. Higher scores on the GHQ-12 indicate a greater likelihood of probable psychiatric morbidity. Finally, variables were created to indicate year of survey interview and country of survey to investigate whether there was any change in likelihood of participation in cancer-related health behaviours in young people over time and if there were differences between Scotland and England.

### Statistical analysis

Socio-demographic characteristics of NEETs and non-NEETs were compared by regressing NEET status on each of the socio-demographic characteristics in a univariate logistic regression model. Logistic regression was used to model the effect of being NEET on the odds of being a current smoker; heavy drinker; not participating in sport; having eaten less than five portions of fruit or vegetables the day before survey interview and having an unhealthy BMI, before and after adjustment for the other independent variables. NEET status was included in the model even if the effect was not significant. Since health behaviours of UE NEETs and EI NEETs are likely to be different, logistic regressions for health behaviours were performed before and after exclusion of EI NEETs. Further, as individuals aged under 18 years are not legally permitted to purchase alcohol or tobacco in Scotland or England, analyses for smoking and alcohol-related outcomes were restricted to survey respondents aged 18 years and over. Missing data were imputed using regression imputation. All analyses were conducted in IBM SPSS Statistics 21 [40].

## Results

### Characteristics of survey respondents

Data were available for 1717 SHeS respondents and 2555 HSE respondents, giving a total sample size of 4,272 respondents. Characteristics of respondents by NEET status are presented in Table 1. In this sample of young people aged 16–24 years, 715 (17%) were classified as being NEET. Of the 715 respondents classified as NEET, 371 (52%) were further defined as unemployed NEETs and 342 (48%) as economically inactive. Two NEETs could not be defined as unemployed or economically inactive due to missing information.

**Table 1:** Characteristics and chi-square analysis of health survey respondents by NEET status

Variable	Categories	All NEETs Included	Economically Inactive NEETs Excluded	Total^a^ (%)
		% NEET	% NEET	
Sex	1 Male	**14	*11	1881 (44)
	2 Female	20	8	2391 (56)
Age^b^		**Mean age NEET = 21	*Mean age NEET = 20	
		Mean age non-NEET = 20	Mean age non-NEET = 20	
Ethnicity	1 White UK & Irish	17	10	3571 (84)
	2 Other (incl. gypsy/traveller)	16	10	684 (16)
Marital status	1 Married/cohabiting	**27	*12	821 (19)
	2 Other (incl. single/separated/divorced)	15	9	3450 (81)
Access to car/van	1 Yes	**11	**6	3024 (71)
	2 No	33	19	1248 (29)
Top academic qualification	1 Degree or higher	**12	**8	536 (13)
	2 HNC/D or equiv (higher education below degree)	12	6	386 (9)
	3 Higher/A-level or equiv (upper school qualification)	9	5	1496 (35)
	4 Standard grade/O-level or equiv (lower school qualification)	21	13	1314 (31)
	5 Foreign or other qualification	34	18	170 (4)
	6 No educational qualification	41	23	347 (8)
Housing tenure	1 Own outright/with mortgage	**8	**5	1955 (46)
	2 Other (incl. part rent/part mortgage, renting, rent-free, squatting)	25	14	2313 (54)
Receipt of means-tested benefits	0 No	**7	**5	1706 (40)
	1 Yes	24	13	2566 (60)
Total annual household income	1 < £15,600	**36	**22	1057 (25)
	2 £15,600-£25,999	17	10	736 (17)
	3 £26,000-£36,399	10	6	510 (12)
	4 £36,400-£51,999	5	3	517 (12)
	5 £52,000-£69,999	5	2	344 (8)
	6 £70,000-£150,000+	7	4	386 (9)
	7 Refused	15	9	426 (10)
	8 Don’t know	14	8	296 (7)
NSSEC	1 Managerial & professional	**6	**4	439 (10)
	2 Intermediate	16	8	652 (15)
	3 Routine & manual	19	11	2262 (53)
	4 Other or never worked/long-term unemployed	18	11	919 (22)
Limiting long-term illness	1 Limiting long-term illness	**32	13	446 (10)
	2 Non-limiting long-term illness	14	8	409 (10)
	3 No limiting long-term illness	15	9	3411 (80)
Self-assessed general health	1 Very good/good	**15	**9	3757 (88)
	2 Fair/bad/very bad	34	19	512 (12)
GHQ-12 score	0 Score 0	**15	**8	2250 (53)
	1 Score 1–2	16	9	1144 (27)
	2 Score 3–4	19	11	476 (11)
	3 Score 5–12	29	19	402 (9)
Survey	1 Health Survey for England (HSE)	17	9	2555 (60)
	2 Scottish Health Survey (SHeS)	17	10	1717 (40)
Survey year	1 2010	18	9	1502 (35)
	2 2011	17	9	1527 (36)
	3 2012	16	10	1243 (29)

*p < 0.05

**p < 0.001

^a^The value for Total may not exactly equal the sum of NEET and non-NEET counts due to missing data within categories. Frequencies and percentages in this column are based on all NEETs

^b^Mean age at survey interview between NEETs and non-NEETs was compared using a 2-sample *t*-test. 95% confidence interval for mu(Age_non-neet_)-mu(Age_neet_) was (−1.2, −0.8) when all NEETs were included and (−0.9, −0.3) when excluding economically inactive NEETs

### Socio-demographic characteristics of NEETs

Results from the univariate logistic regression (Table 2) showed that, before exclusion of economically inactive NEETs, NEETs were significantly more likely (*p <* 0.001) to be female; older in age; be married or cohabiting with a partner; have no car/van access; be educated only to standard grade level or below or, have foreign or no qualifications; not own their home either outright or with a mortgage; receive means-tested benefits; have lower total annual household income; have NS-SEC category other than managerial/professional; have a limiting long-term illness; have fair-bad self-assessed general health and a GHQ-12 score of three or more. There was no difference in the odds of being NEET by ethnicity or between Scottish and English health survey respondents and the odds of being NEET was not significantly different across the three survey years (*p =* 0.843 and *p =* 0.505 respectively). When EI NEETs were excluded, limiting long-term illness was no longer significantly associated with the odds of being NEET (*p =* 0.085) and the direction of the effect of sex on NEET status was reversed with males significantly more likely to be NEET. The direction of the effects of survey (SHeS/HSE) and survey year was also reversed when economically inactive NEETs were excluded; however, the effects of these variables remained non-significant (*p =* 0.166 and *p =* 0.634 respectively). Parameter estimates for remaining variables were either similar or attenuated compared to those before exclusion of EI NEETs.

**Table 2:** Results^a^ from univariate logistic regressions for the effect of socio-demographic characteristics on NEET status

Variable	All NEETs Included	Economically Inactive NEETs Excluded
	OR (95% CI)	OR (95% CI)
Sex		
Male	1.00**	1.00*
Female	1.51 (1.28–1.78)	0.72 (0.58–0.90)
Age	1.16** (1.12–1.20)	1.09** (1.05–1.14)
Ethnicity		
White UK & Irish	1.00	1.00
Other (incl. gypsy/traveller)	0.94 (0.75–1.17)	1.00 (0.75–1.34)
Marital status		
Married/cohabiting	2.13 (1.78–2.56)	1.37 (1.06–1.78)
Other (incl. single/separated/divorced)	1.00**	1.00*
Access to car/van		
Yes	1.00**	1.00**
No	4.13 (3.50–4.88)	3.54 (2.85–4.41)
Top academic qualification		
Degree or higher	1.00**	1.00**
HNC/D or equiv (higher education below degree)	1.00 (0.66–1.50)	0.69 (0.40–1.18)
Higher/A-level or equiv (upper school qualification)	0.77 (0.56–1.06)	0.64 (0.43–0.94)
Standard grade/O-level or equiv (lower school qualification)	1.99 (1.48–2.68)	1.59 (1.11–2.28)
Foreign or other qualification	3.77 (2.48–5.71)	2.48 (1.45–4.25)
No educational qualification	5.18 (3.68–7.28)	3.22 (2.10–4.94)
Housing tenure		
Own outright/with mortgage	0.26 (0.22–0.32)	0.33 (0.26–0.42)
Other (incl. part rent/part mortgage, renting, rent-free, squatting)	1.00**	1.00**
Receipt of means-tested benefits		
No	0.26 (0.21–0.32)	0.34 (0.27–0.44)
Yes	1.00**	1.00**
Total annual household income		
< £15,600	1.00**	1.00**
£15,600-£25,999	0.37 (0.29–0.46)	0.39 (0.29–0.53)
£26,000-£36,399	0.21 (0.15–0.29)	0.21 (0.14–0.33)
£36,400-£51,999	0.09 (0.06–0.14)	0.13 (0.08–0.21)
£52,000-£69,999	0.10 (0.06–0.16)	0.08 (0.04–0.17)
£70,000-£150,000+	0.14 (0.09–0.21)	0.16 (0.10–0.27)
Refused	0.32 (0.23–0.42)	0.35 (0.23–0.51)
Don’t know	0.29 (0.20–0.41)	0.33 (0.21–0.52)
NSSEC		
Managerial & professional	1.00**	1.00**
Intermediate	3.04 (1.94–4.75)	2.23 (1.22–4.06)
Routine & manual	3.69 (2.45–5.56)	3.38 (1.98–5.77)
Other or never worked/long-term unemployed	3.36 (2.18–5.17)	2.24 (1.85–5.68)
Limiting long-term illness		
Limiting long-term illness	2.53 (2.02–3.16)	1.42 (1.01–1.99)
Non-limiting long-term illness	0.91 (0.68–1.22)	0.88 (0.60–1.29)
No limiting long-term illness	1.00**	1.00
GHQ-12 score		
Score 0	1.00**	1.00**
Score 1–2	1.08 (0.89–1.32)	1.07 (0.82–1.39)
Score 3–4	1.37 (1.06–1.77)	1.44 (1.03–2.01)
Score 5–12	2.31 (1.80–2.95)	2.53 (1.85–3.46)
Self-assessed general health		
Very good/good	1.00**	1.00**
Fair/bad/very bad	2.99 (2.43–3.67)	2.43 (1.84–3.20)
Survey		
Health Survey for England (HSE)	1.00	1.00
Scottish Health Survey (SHeS)	0.98 (0.84–1.16)	1.16 (0.94–1.44)
Survey year		
2010	1.00	1.00
2011	0.93 (0.77–1.12)	1.02 (0.79–1.32)
2012	0.89 (0.73–1.09)	1.13 (0.87–1.47)

*p < 0.05

**p < 0.001

^a^Odds ratios (OR) and 95% confidence intervals (95% CI) are presented

### Health outcomes by NEET status

Frequencies and column percentages of participation in cancer-related unhealthy behaviours by NEET status before and after exclusion of economically inactive NEETs are presented in Table 3 with results from logistic regressions of investigating the effect of NEET status on health outcomes in Tables 4, 5, 6, 7 and 8.

**Table 3:** Frequencies (column %) of participation in unhealthy behaviours by NEET status

Outcome	All NEETs Included		Economically Inactive NEETs Excluded		Total (%)	
	NEET (%)	non-NEET (%)	NEET (%)	non-NEET (%)	All NEETs Included	Economically Inactive NEETs Excluded
Current smoker	297 (46)	684 (27)	146 (46)	684 (27)	981 (30)	830 (29)
Heavy drinker^a^	128 (20)	656 (25)	75 (24)	656 (25)	784 (24)	731 (25)
No participation in sport	330 (46)	1003 (29)	143 (38)	1003 (29)	1333 (32)	1146 (30)
Less than 5 portions fruit/vegetables^b^	579 (81)	2652 (76)	307 (82)	2652 (76)	3231 (77)	2959 (77)
Unhealthy BMI^c^	387 (54)	1498 (43)	199 (53)	1498 (43)	1885 (45)	1697 (44)

^a^Heavy drinking refers to consuming >14 units of alcohol per week for females and >21 units for males ^b^Refers to whether the respondent reported eating less than 5 portions of fruit or vegetables the day before survey interview (yes/no)

^c^Refers to whether the respondent was defined as having an unhealthy BMI (yes/no). Unhealthy BMI refers to being ‘underweight’ (BMI <18.5 kg/m^2^); ‘overweight’ (BMI 25–29.99 kg/m^2^) or ‘obese’ (BMI > =30 kg/m^2^)

**Table 4:** Odds ratios and 95% CIs for effect of NEET status on Current Smoking^a,b^

Variable	All NEETs Included	Economically Inactive NEETs Excluded
	OR (95% CI)	OR (95% CI)
UNIVARIATE^c^		
NEET		
No	1.00**	1.00**
Yes	2.38 (1.99–2.84)	2.34 (1.85–2.96)
FULLY ADJUSTED^c^		
NEET		
No	1.00*	1.00*
Yes	1.36 (1.10–1.68)	1.49 (1.14–1.94)
Sex		
Male	1.26 (1.07–1.49)	1.23 (1.03–1.47)
Female	1.00*	1.00*
Age	1.06* (1.01–1.11)	1.06* (1.01–1.11)
Ethnicity		
White UK & Irish	1.00**	1.00*
Other (incl. gypsy/traveller)	0.57 (0.45–0.72)	0.65 (0.50–0.84)
Access to car/van		
Yes	1.00**	1.00**
No	1.56 (1.29–1.89)	1.41 (1.15–1.73)
Top academic qualification		
Degree or higher	1.00**	1.00**
HNC/D or equiv (higher education below degree)	1.75 (1.24–2.46)	1.71 (1.21–2.43)
Higher/A-level or equiv (upper school qualification)	1.39 (1.04–1.86)	1.29 (0.96–1.73)
Standard grade/O-level or equiv (lower school qualification)	2.68 (1.99–3.61)	2.52 (1.85–3.42)
Foreign or other qualification	2.98 (1.91–4.63)	3.47 (2.15–5.60)
No educational qualification	3.04 (2.05–4.51)	3.42 (2.19–5.32)
Housing tenure		
Own outright/with mortgage	1.00**	1.00**
Other (incl. part rent/part mortgage, renting, rent-free, squatting)	1.97 (1.62–2.39)	1.95 (1.59–2.39)
NSSEC		
Managerial & professional	1.00**	1.00**
Intermediate	1.24 (0.90–1.71)	1.28 (0.92–1.77)
Routine & manual	1.38 (1.04–1.84)	1.34 (1.01–1.79)
Other or never worked/long-term unemployed	0.58 (0.39–0.87)	0.52 (0.33–0.80)
Self-assessed general health		
Very good/good	1.00**	1.00**
Fair/bad/very bad	1.97 (1.57–2.47)	2.09 (1.63–2.68)

*p < 0.05

**p < 0.001

^a^The outcome is whether the respondent reported being a current smoker (yes/no)

^b^As the legal minimum age for buying tobacco in Scotland and England is 18 years of age, 16 and 17 year-olds have been excluded from analysis

^c^Univariate refers to the model containing NEET status only and fully adjusted is the model containing all significant socio-demographic and health-related characteristics

**Table 5:** Odds ratios and 95% CIs for effect of NEET status on Heavy Drinking^a,b^

Variable	All NEETs Included	Economically Inactive NEETs Excluded
	OR (95% CI)	OR (95% CI)
UNIVARIATE^c^		
NEET		
No	1.00*	1.00
Yes	0.73 (0.59–0.90)	0.90 (0.69–1.19)
FULLY ADJUSTED^c^		
NEET		
No	1.00*	1.00
Yes	0.71 (0.56–0.91)	0.91 (0.68–1.22)
Sex		
Male	0.71 (0.60–0.85)	0.69 (0.58–0.82)
Female	1.00**	1.00**
Ethnicity		
White UK & Irish	1.00**	1.00**
Other (incl. gypsy/traveller)	0.43 (0.33–0.56)	0.41 (0.31–0.54)
Access to car/van		
Yes	1.00*	1.00*
No	1.24 (1.03–1.49)	1.29 (1.06–1.56)
Marital status		
Married/cohabiting	0.65 (0.53–0.80)	0.67 (0.54–0.83)
Other (incl. single/separated/divorced)	1.00**	1.00**
Receipt of means-tested benefits		
No	1.00*	1.00*
Yes	0.80 (0.67–0.95)	0.80 (0.67–0.96)
NSSEC		
Managerial & professional	1.00*	1.00*
Intermediate	1.06 (0.78–1.45)	1.07 (0.78–1.46)
Routine & manual	1.40 (1.08–1.83)	1.40 (1.07–1.84)
Other or never worked/long-term unemployed	1.13 (0.80–1.61)	1.18 (0.82–1.70)

*p < 0.05

**p < 0.001

^a^The outcome is whether the respondent was defined as being a heavy drinker (yes/no). Heavy drinking refers to consuming >14 units of alcohol per week for females and >21 units for males

^b^As the legal minimum age for buying alcohol in Scotland and England is 18 years of age, 16 and 17 year-olds have been excluded from analysis

^c^Univariate refers to the model containing NEET status only and fully adjusted is the model containing all significant socio-demographic and health-related characteristics

**Table 6:** Odds ratios and 95% CIs for effect of NEET status No Participation in Sport^a^

Variable	All NEETs Included	Economically Inactive NEETs Excluded
	OR (95% CI)	OR (95% CI)
UNIVARIATE^b^		
NEET		
No	1.00**	1.00**
Yes	2.12 (1.80–2.50)	1.54 (1.23–1.92)
FULLY ADJUSTED^b^		
NEET		
No	1.00**	1.00*
Yes	1.52 (1.26–1.82)	1.30 (1.03–1.65)
Sex		
Male	0.65 (0.57–0.75)	0.67 (0.58–0.77)
Female	1.00**	1.00**
Age	1.04* (1.01–1.07)	1.04* (1.01–1.07)
Ethnicity		
White UK & Irish	1.00*	
Other (incl. gypsy/traveller)	1.22 (1.02–1.47)	
Access to car/van		
Yes	1.00**	1.00*
No	1.31 (1.13–1.53)	1.29 (1.10–1.52)
Top academic qualification		
Degree or higher	1.00**	1.00**
HNC/D or equiv (higher education below degree)	1.62 1.18–2.23()	1.54 (1.11–2.13)
Higher/A-level or equiv (upper school qualification)	1.84 (1.42–2.37)	1.76 (1.36–2.28)
Standard grade/O-level or equiv (lower school qualification)	2.29 (1.76–2.99)	2.22 (1.69–2.91)
Foreign or other qualification	2.77 (1.87–4.10)	2.79 (1.82–4.26)
No educational qualification	2.49 (1.79–3.46)	2.41 (1.68–3.46)
Limiting long-term illness		
Limiting long-term illness	1.22 (0.96–1.54)	
Non-limiting long-term illness	0.81 (0.64–1.03)	
No limiting long-term illness	1.00*	
Self-assessed general health		
Very good/good	1.00*	1.00*
Fair/bad/very bad	1.44 (1.15–1.79)	1.39 (1.11–1.73)
Survey year		
2010	1.00*	1.00*
2011	0.94 (0.81–1.10)	0.91 (0.77–1.08)
2012	0.79 (0.67–0.94)	0.75 (0.63–0.90)
Survey		
Health Survey for England		1.00*
Scottish Health Survey		0.84 (0.72–0.97)

*p < 0.05

**p < 0.001

^a^The outcome is whether the respondent reported no participation in sporting activities (yes/no)

^b^Univariate refers to the model containing NEET status only and fully adjusted is the model containing all significant socio-demographic and health-related characteristics

**Table 7:** Odds ratios and 95% CIs for effect of NEET status on Fruit & Vegetable Consumption^a^

Variable	All NEETs Included	Economically Inactive NEETs Excluded
	OR (95% CI)	OR (95% CI)
UNIVARIATE^b^		
NEET		
No	1.00*	1.00*
Yes	1.34 (1.09–1.64)	1.46 (1.11–1.93)
FULLY ADJUSTED^b^		
NEET		
No	1.00	1.00
Yes	1.10 (0.87–1.39)	1.23 (0.91–1.66)
Fruit consumption the same as usual		
Less than usual	1.00**	1.00**
More than usual	0.24 (0.18–0.32)	0.24 (0.18–0.32)
About the same as usual	0.41 (0.34–0.50)	0.39 (0.32–0.48)
Vegetable consumption the same as usual		
Less than usual	1.00**	1.00**
More than usual	0.71 (0.55–0.92)	0.74 (0.56–0.96)
About the same as usual	0.59 (0.50–0.70)	0.59 (0.49–0.70)
Sex		
Male	1.19 (1.02–1.39)	1.19 (1.02–1.40)
Female	1.00*	1.00*
Ethnicity		
White UK & Irish	1.00**	1.00**
Other (incl. gypsy/traveller)	0.61 (0.51–0.75)	0.64 (0.52–0.79)
Top academic qualification		
Degree or higher	1.00**	1.00**
HNC/D or equiv (higher education below degree)	1.24 (0.90–1.70)	1.24 (0.89–1.71)
Higher/A-level or equiv (upper school qualification)	1.22 (0.96–1.54)	1.18 (0.92–1.50)
Standard grade/O-level or equiv (lower school qualification)	1.97 (1.51–2.56)	1.90 (1.45–2.49)
Foreign or other qualification	1.89 (1.20–2.96)	1.95 (1.20–3.19)
No educational qualification	1.51 (1.07–2.14)	1.45 (0.99–2.12)
Receipt of means-tested benefits		
No	1.00*	1.00*
Yes	1.19 (1.00–1.40)	1.20 (1.01–1.43)
Total annual household income		
< £15,600	1.00*	1.00*
£15,600-£25,999	0.87 (0.68–1.12)	0.92 (0.70–1.20)
£26,000-£36,399	1.06 (0.79–1.41)	1.14 (0.85–1.55)
£36,400-£51,999	0.81 (0.61–1.07)	0.88 (0.66–1.17)
£52,000-£69,999	0.68 (0.50–0.93)	0.74 (0.54–1.02)
£70,000-£150,000+	0.57 (0.43–0.76)	0.62 (0.46–0.84)
Refused	0.88 (0.66–1.17)	0.98 (0.72–1.32)
Don’t know	0.85 (0.62–1.18)	0.99 (0.70–1.40)
Self-assessed general health		
Very good/good	1.00*	1.00*
Fair/bad/very bad	1.34 (1.03–1.73)	1.34 (1.01–1.77)
Survey		
Health Survey for England (HSE)	1.00**	1.00**
Scottish Health Survey (SHeS)	1.82 (1.54–2.16)	1.83 (1.53–2.18)
Survey year		
2010	1.00**	1.00**
2011	1.08 (0.90–1.29)	1.09 (0.90–1.31)
2012	1.59 (1.28–1.97)	1.56 (1.25–1.94)

*p < 0.05

**p < 0.001

^a^The outcome is whether the respondent reported eating less than 5 portions of fruit or vegetables the day before survey interview (yes/no)

^b^Univariate refers to the model containing NEET status only and fully adjusted is the model containing all significant socio-demographic and health-related characteristics

**Table 8:** Odds ratios and 95% CIs for effect of NEET status on BMI^a^

Variable	All NEETs Included	Economically Inactive NEETs Excluded
	OR (95% CI)	OR (95% CI)
UNIVARIATE^b^		
NEET		
No	1.00**	1.00**
Yes	1.57 (1.33–1.84)	1.62 (1.23–1.88)
FULLY ADJUSTED^b^		
NEET		
No	1.00**	1.00*
Yes	1.36 (1.15–1.61)	1.34 (1.07–1.68)
Age	1.05** (1.03–1.09)	1.07** (1.03–1.10)
Ethnicity		
White UK & Irish		1.00*
Other (incl. gypsy/traveller)		0.81 (0.68–0.98)
Marital status		
Married/cohabiting	1.29 (1.09–1.53)	1.22 (1.01–1.48)
Other (incl. single/separated/divorced)	1.00*	1.00*
Top academic qualification		
Degree or higher		1.00*
HNC/D or equiv (higher education below degree)		1.31 (0.99–1.74)
Higher/A-level or equiv (upper school qualification)		1.17 (0.93–1.46)
Standard grade/O-level or equiv (lower school qualification)		1.42 (1.11–1.81)
Foreign or other qualification		1.56 (1.04–2.31)
No educational qualification		1.54 (1.11–2.14)
NSSEC		
Managerial & professional		1.00*
Intermediate		1.03 (0.97–1.33)
Routine & manual		0.80 (0.64–1.00)
Other or never worked/long-term unemployed		0.94 (0.71–1.24)
Self-assessed general health		
Very good/good	1.00**	1.00**
Fair/bad/very bad	1.51 (1.24–1.82)	1.56 (1.26–1.93)

*p < 0.05

**p < 0.001

^a^The outcome is whether the respondent was defined as having an unhealthy BMI (yes/no). Unhealthy BMI refers to being ‘underweight’ (BMI <18.5 kg/m^2^); ‘overweight’ (BMI 25–29.99 kg/m^2^) or ‘obese’ (BMI > =30 kg/m^2^)

^b^Univariate refers to the model containing NEET status only and fully adjusted is the model containing all significant socio-demographic and health-related characteristics

Results from Table 4 demonstrated that NEETs were significantly more likely to be current smokers than non-NEETs (*p <* 0.001) both before (odds ratio (OR) = 2.38, 95% confidence interval (CI) = 1.99-2.84) and after (OR = 2.34, 95% CI = 1.85-2.96) EI NEETs were excluded (i.e. when considering UE NEETs only). This result persisted even after adjustment for significant socio-demographic and health-related confounders. Odds ratios for heavy drinking (Table 5) demonstrated that NEETs were less likely to be heavy drinkers than non-NEETs; however, this decreased risk was only significant before exclusion of EI NEETs (OR = 0.73, 95% CI = 0.59-0.90). Adjusting for significant socio-demographic characteristics did not alter results. NEETs were significantly more likely to report not taking part in sporting activities (Table 6) (OR = 2.12, 95% CI = 1.80-2.50 when all NEETs were included). The effect was attenuated, but still significant, after excluding EI NEETs (OR = 1.54, 95% CI = 1.23-1.92). The increased risk of reporting not taking part in any sporting activities amongst NEETs remained significant even in the fully-adjusted model. The likelihood of reporting non-participation in sporting activities significantly decreased over time (*p <* 0.05) as demonstrated by the odds ratios for survey year.

NEETs were significantly more likely to report not having eaten the UK Government-recommended five portions of fruit or vegetables the day before survey interview (*p <* 0.05) than non-NEETs both before (OR = 1.34, 95% CI = 1.09-1.64) and after (OR = 1.46, 95% CI = 1.11-1.93) exclusion of EI NEETs (Table 7). Results were attenuated in the fully-adjusted model and the increased risk was no longer significant both before or exclusion of EI NEETs. The odds of reporting not having eaten at least five portions significantly increased over time (*p <* 0.001) as demonstrated by the odds ratios for survey year.

Finally, NEETs were also highly significantly more likely to have an ‘unhealthy’ BMI (*p <* 0.001) than non NEETs both before (OR = 1.57, 95% CI = 1.33–1.84) and after (OR = 1.62, 95% CI = 1.23–1.88) excluding EI NEETs (Table 8). The increased risk amongst NEETs remained significant even in the fully-adjusted model, but the effect was slightly stronger before exclusion of EI NEETs.

### Health outcomes by survey region

No participation in sport and fruit and vegetable consumption were the only outcomes for which there was a significant effect of country of survey. However, survey region was only significant (*p <* 0.05) after excluding EI NEETs for the ‘no participation in sport’ outcome (Table 6). In the fully-adjusted model, respondents to the SHeS were significantly less likely to report no participation in sport than respondents to the HSE (OR = 0.84, 95% CI = 0.72–0.97) after exclusion of EI NEETs. For fruit and vegetable consumption (Table 7), respondents to the SHeS were significantly more likely to report not having eaten at least five portions (*p <* 0.001 before and after exclusion of EI NEETs) compared to respondents to HSE.

## Discussion

This study aimed to contribute to the limited evidence base on whether not being in education, employment or training was associated with a greater likelihood of participating in cancer-related behaviours.

### Socio-demographic characteristics of NEETs

Increasing age was significantly associated with increased odds of being NEET; however, this effect was stronger before exclusion of EI NEETs. This result, along with females being at an increased risk of being NEET before excluding EI NEETs, possibly reflects females taking time out of education or employment to start a family as they get older. The fact that effects of gender and age are reversed or attenuated when EI NEETs are excluded would appear to support this belief. Findings also support previous reports of NEETs being from socioeconomically-disadvantaged backgrounds [41].

In terms of health characteristics, fair-bad self-assessed general health and short-term non-psychotic psychiatric morbidity, (GHQ-12 score), were significantly associated with an increased risk of being NEET. These are known indicators of poorer mental health, which has been previously associated with becoming NEET [15].

Other differences noted between EI and UE NEETs related to having a limiting long-term illness, which was no longer significant after excluding EI NEETs. This result is expected since excluding EI NEETs would remove individuals with long-term illness/disability. Further, there was an increased likelihood of being NEET amongst SHeS versus HSE respondents after excluding EI NEETs. Although the difference was not statistically significant, this would suggest greater rates of unemployment amongst young people in Scotland compared to England. Increasing the sample size by adding data from more recent health surveys as they become available may confirm significant differences in the likelihood of being NEET across different regions of the UK.

### Cancer-related health behaviours of NEETs

This study found a greater tendency for NEETs to participate in cancer-related unhealthy behaviours compared to non-NEETs. However, there were some differences in the effect of NEET status before and after exclusion of EI NEETs.

There are several possible explanations as to why participation in unhealthy behaviours may be greater in NEETs compared to non-NEETs. As confirmed by this study and in previous studies, NEETs are more likely to be poorly educated [42]. Poor education may diminish knowledge of how to live a healthy life [43] and reduce decision-making abilities for making healthy choices [44]. However, there remained an independent effect of NEET status on participation on some unhealthy behaviours even after adjustment for top academic qualification. Similarly, as demonstrated in this study and in previous research, NEETs were more likely to have reduced income [41]. Reduced income may restrict healthy dietary options or the ability to participate in healthy recreational activities [45]. This could explain associations between being NEET and reduced fruit and vegetable consumption, participation in sport and an unhealthy BMI. Indeed, in addition to other socio-demographic and health-related confounders, total annual household income explained the effect of NEET status on fruit and vegetable consumption before and after EI NEETs were excluded. However, being NEET remained independently associated with reduced participation in sport and an unhealthy BMI even after adjustment for total annual household income. Being NEET also remained significantly associated with being a current smoker after adjustment for total annual household income. It could be expected that reduced income may lead to decreased participation in unhealthy behaviours such as smoking and drinking due to the financial cost associated with these behaviours, but there is a well-known link between unemployment and smoking in young people [46]. As well as the addiction to nicotine, smoking may be a coping mechanism as a way of dealing with the stresses associated with unemployment [46–48]. Indeed, the association between NEET status and current smoking strengthened when considering unemployed NEETs only by excluding EI NEETs.

An inverse association between NEET status and heavy drinking was observed when considering all NEETs; however, this association became non-significant on exclusion of EI NEETs. Available socio-demographic and health-related significant risk factors could not explain the negative association between being NEET and heavy drinking when considering all NEETs. For example, although some previous research has reported lower levels of alcohol consumption amongst young mothers [16, 49] and young people who are disabled [50], adjusting for gender did not alter the effect of NEET status on heavy drinking and limiting long-term illness was not significantly associated with heavy drinking in this study. However, this study did not control for whether respondents had children. Early parenthood has been shown to moderate alcohol consumption [49]. As the likelihood of having children may be greater amongst young people who are economically inactive, particularly as analysis of the heavy drinking outcome is restricted to those aged 18 and over, the NEET status variable, before exclusion of EI NEETs, may be accounting for residual confounding resulting from not controlling for whether the respondent had children.

The effect of NEET status on reporting of no participation in sport was also attenuated on exclusion of EI NEETs, but the effect remained significant. Again, this result is not unexpected given previous reports of lower levels of sport amongst young mothers [51] and young people who are permanently or temporarily sick, disabled or injured [52]. Although physical inactivity has been linked with certain cancers [24], the ‘no participation in sport’ measure used in this study only reflects one dimension of physical inactivity. Other aspects of physical activity (PA), such as occupational-, transport- and domestic-related domains, which, to some extent, have also been shown to be protective for health [24], including some cancers [53, 54], have not been considered here. Using a more comprehensive measure of PA may have altered the effect of NEET status on this outcome. In particular, the observed attenuation in the effect of NEET status after excluding EI NEETs may have been smaller if a measure of occupational-related activity had also been included. Alternatively, if a domestic-related measure of PA had been included then there could plausibly have been a greater attenuation of the effect of NEET status after excluding EI NEETs as a result of excluding young, economically inactive females. Such individuals may be exposed to higher levels of PA through the physical demands of looking after the home and young family.

The association between being NEET and reduced fruit and vegetable consumption was stronger when considering UE NEETs only. Although both unemployment and disability have been associated with an increased risk of food poverty, including insufficient consumption of fruit and vegetables [55], this finding would suggest that the risk is greater in unemployed young people than those who are economically inactive when compared to their ‘non-NEET’ peers. However, the effect of NEET status on this outcome could be explained by socio-demographic and health-related confounders both before and after exclusion of EI NEETs.

The increase in reporting of not eating at least five portions of fruit and vegetables over time was interesting given there had been a decrease in reporting of non-participation in sport over time. It would therefore appear that this study does not support previously-reported associations between sedentary behaviours and less healthy eating patterns in young adults [56]. However, the increase in participation in sport in 2012 found in this study coincides with London hosting the 2012 Olympic Games. The recognised peak in participation in sport in 2012 has since declined [57], therefore findings may not have been similar if more recent data had been used. Since the decline has been shown to be greater amongst more socioeconomically-disadvantaged groups [58], NEETs may be more vulnerable to this decline.

### Strengths and limitations

Merging of health surveys allowed for cross-national comparisons of health behaviours associated with cancer. A particular strength was that the comprehensive data on economic profiles of respondents collected by these surveys allowed NEETs to be classed further as EI NEETs or UE NEETs. This was important since previous studies reported differences in socio-demographic characteristics and health behaviours of EI and UE NEETs [59, 60]; a finding that was also confirmed by this study.

A strength of using the Scottish Health Survey is that information on respondents can be further linked to Scottish Morbidity Records, including the Scottish Cancer Registry (SMR06). The SMR06 scheme collects information on all residents in Scotland that have had a diagnosis of cancer. Future research could investigate the role of NEET status on developing cancer after adjustment for cancer-related behaviours and other socio-demographic characteristics, for a subset of the participants used in this study.

Cancer-related health behaviours, such as smoking, have also been associated with a greater risk of becoming NEET [15]. Due to the cross-sectional nature of the data, this study could not determine the temporal sequence of becoming NEET and participating in cancer-related behaviours and it was not possible to determine a causal effect of being NEET on cancer-related health behaviours, therefore reverse causation is possible.

## Conclusions

This study has shown that NEETs were at an increased risk of exhibiting cancer-related behaviours compared to non-NEETs, including smoking, not participating in sport and having an unhealthy BMI. Attempts to reduce participation in such behaviours amongst NEETs may contribute to a reduction in cancers associated with these behaviours. However, policymakers should be aware of differences between unemployed and economically and inactive NEETs. This was particularly relevant for heavy drinking. As the likelihood of becoming NEET is greater in socioeconomically-disadvantaged groups, interventions to tackle unhealthy behaviours among NEETs may contribute to a reduction in health inequalities.

## Abbreviations

CI: Confidence interval
EI: Economically inactive
GHQ-12: 12-item general health questionnaire
HSE: Health survey for england
NEET: Not in education employment or training
NS-SEC: National statistics socio-economic classification
OR: Odds ratio
SHeS: Scottish health survey
UE: Unemployed
